# Phosphodiesterase 5 (PDE5) Is Highly Expressed in Cancer-Associated Fibroblasts and Enhances Breast Tumor Progression

**DOI:** 10.3390/cancers11111740

**Published:** 2019-11-06

**Authors:** Stefania Catalano, Salvatore Panza, Giuseppina Augimeri, Cinzia Giordano, Rocco Malivindi, Luca Gelsomino, Stefania Marsico, Francesca Giordano, Balázs Győrffy, Daniela Bonofiglio, Sebastiano Andò, Ines Barone

**Affiliations:** 1 Department of Pharmacy, Health and Nutritional Sciences, University of Calabria, 87036 Rende (CS), Italy; stefcatalano@libero.it (S.C.); sasapanza@libero.it (S.P.); giusy.augimeri@gmail.com (G.A.); cinzia.giordano@unical.it (C.G.); rocco.malivindi@unical.it (R.M.); lugelso@gmail.com (L.G.); stefania.marsico@unical.it (S.M.); francesca.giordano@unical.it (F.G.); daniela.bonofiglio@unical.it (D.B.); 2 Centro Sanitario, University of Calabria, 87036 Rende (CS), Italy; 3 MTA TTK Lendület Cancer Biomarker Research Group, Semmelweis University 2nd Dept. of Pediatrics, 1094 Budapest, Hungary; zsalab2@yahoo.com

**Keywords:** breast cancer, PDE5, tumor microenvironment, targeted therapy, CXCL16

## Abstract

The overexpression of phosphodiesterase (PDE) 5 is frequently found in various human cancers, such as those of the breast. However, PDE5’s role in the tumor microenvironment is still unknown. As PDE5 represents a high-value therapeutic target, we investigated whether the expression and function of PDE5 in breast cancer-associated fibroblasts (CAFs) may be clinically relevant to malignant progression. PDE5 expression was increased in human breast cancer stroma compared with normal stroma and was correlated to a shorter overall survival. Treatment of CAFs, isolated from breast tumor biopsies, with selective PDE5 inhibitors inhibited their proliferation, motility, and invasiveness, and negatively controlled tumor–stroma interactions in both ‘in vitro’ and ‘in vivo’ models. PDE5 stable overexpression transformed immortalized mouse embryonic fibroblasts (MEFs) towards an activated fibroblast phenotype, impacting their intrinsic characteristics and paracrine effects on breast cancer cell growth and migration through an enhanced production of the C-X-C motif chemokine 16 (CXCL16). On the other hand, CAF exposure to PDE5 inhibitors was associated with reduced CXCL16 expression and secretion. Importantly, CXCL16 levels in breast cancer stroma showed a strong correlation with PDE5 levels and poor patient outcomes. In conclusion, PDE5 is overexpressed in breast cancer stroma, enhances the tumor-stimulatory activities of fibroblasts, and impacts clinical outcomes; thus, we propose this enzyme as an attractive candidate for prognosis and a potential target for treatments in breast cancer patients.

## 1. Introduction

Breast cancer is the most commonly diagnosed neoplasia among women worldwide, accounting for 25% of all cancer cases [1]. Despite advances in early detection and adjuvant treatment, about 30% of patients may experience recurrent disease and will eventually die of it. Therefore, there is a critical need for the discovery of adequate therapeutic and prognosis biomarkers to improve clinical outcomes.

In many solid tumors, malignant initiation and progression is associated with the activation of stromal fibroblasts into myofibroblasts or carcinoma-associated fibroblasts (CAFs), characterized by the expression of α-smooth muscle actin (α-SMA) and fibroblast activated protein (FAP) [2]. CAFs are the predominant cellular component within the host stromal constituents and, acting as a motile cohesive unit able to penetrate the cancerous compartment and as a paracrine signaling source niche, possess the abilities to drive tumor cell proliferation, survival, migration and invasion. The key features of CAFs, which distinguish them from their normal counterparts, include their growth characteristic, migratory behavior, and biosynthetic activities, such as altered expression of growth factors and cytokines (e.g., transforming growth factor β (TGFβ), insulin-like growth factor I (IGF-I) and II (IGF-II), leptin, interleukin-6 (IL-6), chemokine (C-C motif) ligand 7 (CCL7), C-X-C motif Chemokine 12 (CXCL12) and 16 (CXCL16)) [3,4,5,6]. However, in spite of these studies, strategies to treat human breast cancer stroma are just beginning to emerge.

Specific phosphodiesterase (PDE)5 inhibitors, such as sildenafil, tadalafil, and vardenafil, are being used in clinical practice to treat erectile dysfunction (ED) as well as pulmonary arterial hypertension (PAH), but these agents have also been identified as one of the most successful cases of drug repurposing because they were initially developed for angina and then repositioned for further disorders [7,8]. In this scenario, recent attention in examining their potential uses as anticancer agents acting with new mechanisms has been drawn, due to the detection of enhanced PDE5 levels in various human tumors in comparison with normal or surrounding non-cancerous tissues [9,10,11,12,13,14]. PDE5 inhibitors are effective, safe, and well-tolerated molecules that act by specifically inhibiting the activity of PDE5, a metallo-hydrolase with the unique function of catalyzing the breakdown of cyclic guanosine monophosphate (cGMP) into its biologically inactive 5′-derivative, thus modulating the amplitude and duration of cGMP levels and the associated biological responses [15]. The signaling mediated by cGMP, via several activated downstream effectors, including PKG (cGMP-dependent protein kinase), cyclic-nucleotide-gated ion channels and/or cyclic adenosine monophosphate (cAMP) pathways, has been reported to function as a tumor suppressor pathway, halting tumor growth, invasiveness, and angiogenesis [16,17,18,19,20]. On the other hand, altered cGMP homeostasis and increased PDE5 expression have been described in several human carcinomas, such as those of the breast. In breast cancer clinical samples, PDE5 overexpression was validated by RT-PCR and immunohistochemistry assays, and was associated with important clinical parameters, such as tumor grading, stage, lymph node positivity, and poor prognosis [11,13,14]. Accordingly, several ‘in vitro’ observations have shown anti-proliferative and pro-apoptotic effects of PDE5 inhibitors [9,10,12,21,22,23,24]. These drugs may also act as chemopreventive agents, due to their ability to suppress 1-methyl-1-nitrosourea (MNU)-induced mammary carcinogenesis [25]. Moreover, it has been demonstrated that the blockade of PDE5, which is mainly expressed in the stromal compartment of the prostate, reduced fibroblast proliferation and reverted fibroblast-to-myofibroblast trans-differentiation of primary prostatic stromal cells, a hallmark of stromal remodeling, highlighting the potential of PDE5 inhibitors to target the stromal compartment [26,27].

Herein, we show for the first time, that PDE5 is overexpressed in breast cancer stroma and plays an important role in pro-neoplastic features of activated fibroblasts through the secretion of CXCL16. Our results identified a novel molecular target able to abrogate stromal–tumor cell interactions and thus attenuate breast tumorigenesis, with important implications in translational oncology.

## 2. Results

### 2.1. Aberrant High PDE5 Expression in the Stroma Is Associated with Breast Cancer Progression

**Figure 1:**
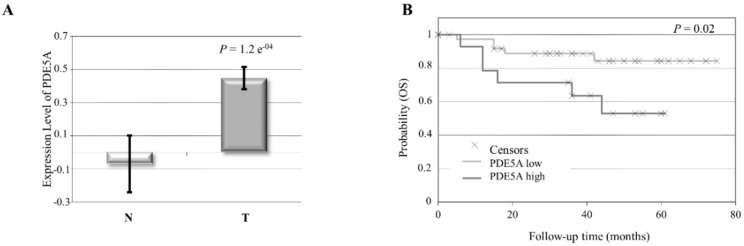
Phosphodiesterase (PDE) 5 expression levels in breast cancer stroma. (**A**) Gene expression levels of PDE5A in normal (N) and breast cancer (T) stroma samples. *p* = 1.2 e^−4^. (**B**) Kaplan–Meier survival analysis relating stromal PDE5 levels and overall survival (OS) in breast cancer patients. *p* = 0.02.

To investigate the role of PDE5 stromal expression in breast cancer, we first analyzed its levels using a Gene Expression Omnibus (GEO) dataset consisting of stroma derived from invasive ductal breast carcinomas and individual-matched normal breast adjacent tissues. PDE5 expression was significantly higher in breast cancer stroma samples as compared to normal ones (Figure 1A). However, its expression did not significantly associate with molecular subtypes, lymph node status, grade, estrogen receptor (ER), progesterone receptor (PR), and HER2 positivity, most probably due to the small number of patients in each subgroup. Importantly, Kaplan–Meier survival analysis showed that increased PDE5 stromal levels were correlated with a statistically significant shorter overall survival of breast cancer patients in respect to tumors expressing low levels of the enzyme in their stroma (Figure 1B), proposing a potential role for stromal PDE5 in breast carcinoma progression.

**Figure 2:**
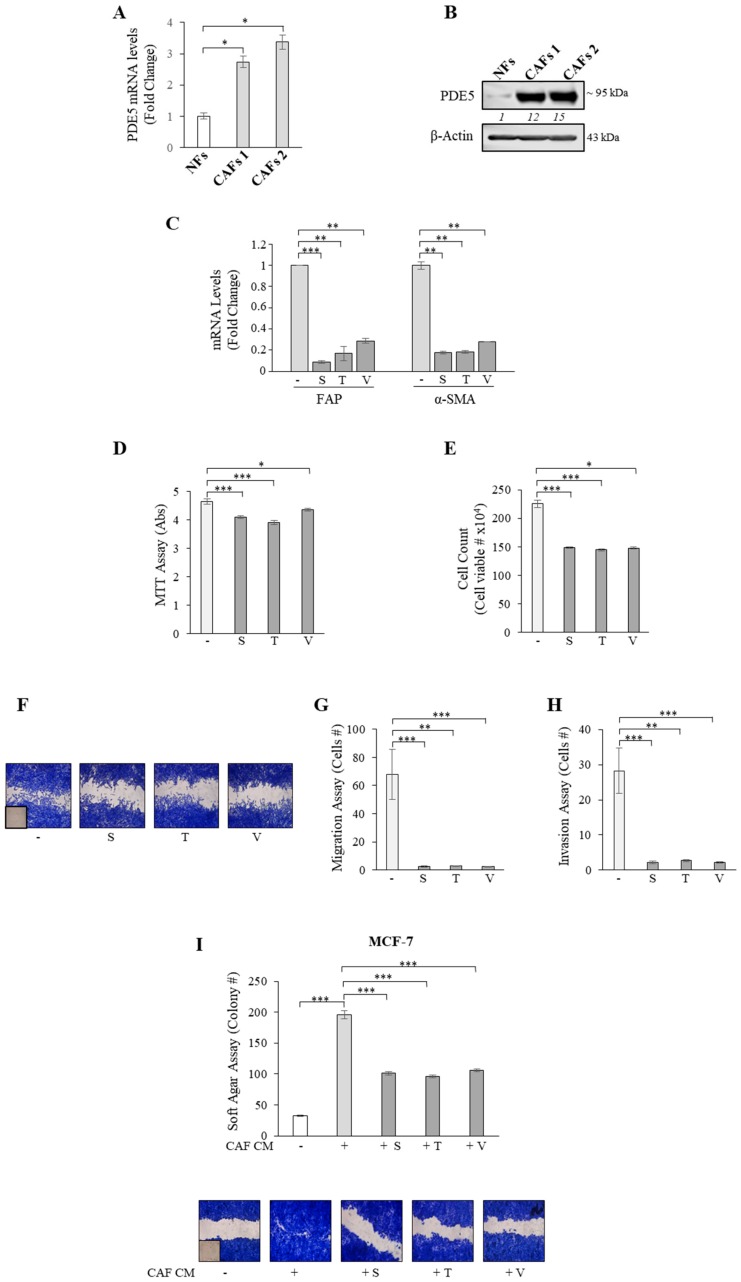
Inhibition of PDE5 activity affects the pro-tumoral features of breast cancer-associated fibroblasts (CAFs). (**A**) Real time RT-PCR assay for PDE5 mRNA expression in normal fibroblasts (NFs) and CAFs 1 and 2. (**B**) Immunoblotting showing PDE5 protein expression. β-Actin was used as a control for equal loading and transfer. Italicized numbers below blots represent the mean of the band optical density expressed as fold over NFs for CAFs 1 and 2. (**C**) Real-time RT-PCR assay for fibroblast activated protein (FAP) and α-smooth muscle actin (α-SMA) mRNA expression in CAFs treated with vehicle (−) or the PDE5 inhibitors: sildenafil (S, 10 µM), tadalafil, (T, 10 µM), and vardenafil (V, 10 µM) for 24 h. (**D**) MTT (3-(4,5-Dimethylthiazol-2-yl)-2,5-Diphenyltetrazolium Bromide) growth and (**E**) Trypan blue cell count assays in CAFs treated as indicated for 48 hours. (**F**) Wound healing assay in CAFs treated as indicated. Inset, time 0. Pictures are representative of three independent experiments. (**G**) Boyden chamber transmigration and (**H**) and invasion assays in CAFs treated as indicated. (**I)** Soft agar growth (upper panel) and wound healing (lower panel) assays in MCF-7 breast cancer cells incubated with conditioned medium (CM) derived from CAFs treated with vehicle (−) or the PDE5 inhibitors (+S, +T, +V) as indicated. Inset, time 0. Pictures are representative of three independent experiments. The values represent the mean ±SEM of three different experiments, each performed in triplicate. * *p* < 0.05, ** *p* < 0.005, *** *p* < 0.0005.

To confirm these clinical observations, fibroblasts were extracted from two human invasive mammary ductal carcinomas (named as CAFs 1 and 2) and characterized on the basis of their long and spindle-shaped morphology and elevated expression of fibroblast activation protein (FAP) and alpha-smooth muscle actin (α-SMA) as compared to normal fibroblasts (NFs) (Supplementary Figure S1). Real-time RT-PCR analysis revealed that PDE5 mRNA expression was higher in CAFs than NFs (Figure 2A). Increased PDE5 expression in CAFs was also confirmed by evaluating protein levels using immunoblotting (Figure 2B). Of relevance, treatment with the PDE5 inhibitors sildenafil, tadalafil, and vardenafil significantly reduced the expression of activated fibroblast markers, such as FAP and α-SMA in CAFs (Figure 2C). Furthermore, inhibition of PDE5 activity resulted in a significant decrease in CAF proliferation (Figure 2D,E), motility (Figure 2F,G), and invasion (Figure 2H). In line with previous findings shown in cancer epithelial cells [14,24], our data also highlighted a major role for PDE5 in controlling migration and invasion processes in stromal cells. We then investigated whether PDE5 may function as a possible regulator of CAF impact on adjacent breast tumor cell proliferation and migration by using co-culture experiments. MCF-7 breast cancer epithelial cells, a commonly used and well characterized ‘in vitro’ model of the most frequent human breast cancer subtype (i.e., luminal A estrogen receptor-positive one), were co-cultured with conditioned medium (CM) derived from CAFs treated with sildenafil, tadalafil, and vardenafil and growth was assessed by soft agar anchorage-independent assay (Figure 2I, upper panel). As expected, colony numbers of MCF-7 breast cancer cells were significantly increased in the presence of CAF-derived CM compared to control medium (-), whereas they were significantly reduced when cells were incubated with CM derived from CAFs treated with PDE5 inhibitors. In the same experimental conditions, sildenafil, tadalafil, and vardenafil negatively affected CAF-mediated increase on MCF-7 cell migration (Figure 2I, lower panel).

**Figure 3:**
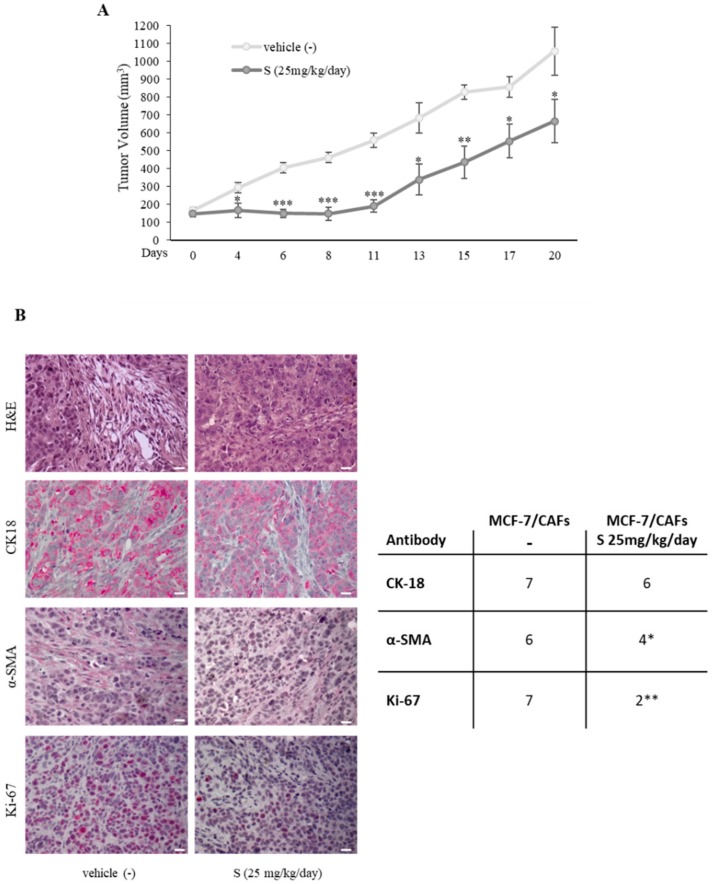
Impact of sildenafil treatment on tumor growth of MCF-7/CAF xenografts. (**A**) MCF-7 cells were co-injected with CAFs subcutaneously into nude mice (8 mice/each group). Vehicle (−) and sildenafil treatment (25 mg/kg/day) were started when tumor size reached 150 mm^3^ (day 0) and delivered daily to the animals by intraperitoneal (i.p.) injection. Tumor volume mean ± SEM is shown. (**B**) Left panel, representative images of hematoxylin and eosin (H&E), human cytokeratin 18 (CK18), α-SMA, and Ki-67 immunohistochemical staining of MCF-7/CAF xenograft tumor sections. Scale bar = 25 µm. Right panel: immunohistochemistry scores. Cases were scored according to Allred immunohistochemistry (IHC) score [28] which includes both the proportion and intensity scores (range from 0 to 8). * *p* < 0.05; ** *p* < 0.005; *** *p* < 0.0005.

Based on these results showing that PDE5 inhibition may thwart tumor-fostering capabilities of CAFs ‘in vitro’, mouse xenograft models were used to investigate sildenafil effects on breast cancer growth ‘in vivo’. To mimic the in vivo conditions where cancer epithelial cells coexist with CAFs within a tumor microenvironment, MCF-7 breast cancer cells were implanted with CAFs into the mouse intrascapular region and tumor growth was observed after the treatment with vehicle or 25 mg/kg/day sildenafil. This administration was well accepted since no modifications in body mass, food/water ingestion, and motor function were detected. Furthermore, after their sacrifice, we did not observe any changes in the weight and the histology of the main organs (i.e., liver, lung, spleen, and kidney) between vehicle- and sildenafil-treated mice, denoting an absence of toxic outcomes at the dose chosen. As presented in Figure 3A, sildenafil treatment induced a significant regression in tumor growth of MCF-7/CAF groups compared to vehicle treatment. Interestingly, hematoxylin and eosin stain revealed an increased infiltration of CAFs into the breast tumor bulk in vehicle-treated mice, but not in sildenafil-treated ones (Figure 3B). Incubation with anti-human Cytokeratin 18 and α-SMA antibodies was performed to confirm the human epithelial and connective origin in examined tissues (Figure 3B). According to previously shown results, α-SMA expression in CAFs was reduced after sildenafil exposure. In addition, we revealed in sildenafil-treated xenograft tumors a significant decrease of protein levels of Ki67, a widely recognized proliferation indicator, in breast cancer epithelial cells (Figure 3B).

Therefore, these data provide, for the first time, evidence that PDE5 is overexpressed in CAFs and could contribute to their tumor-promoting features.

### 2.2. PDE5 Overexpression Activates Fibroblasts towards a Cancer-Associated Fibroblast Phenotype

**Figure 4:**
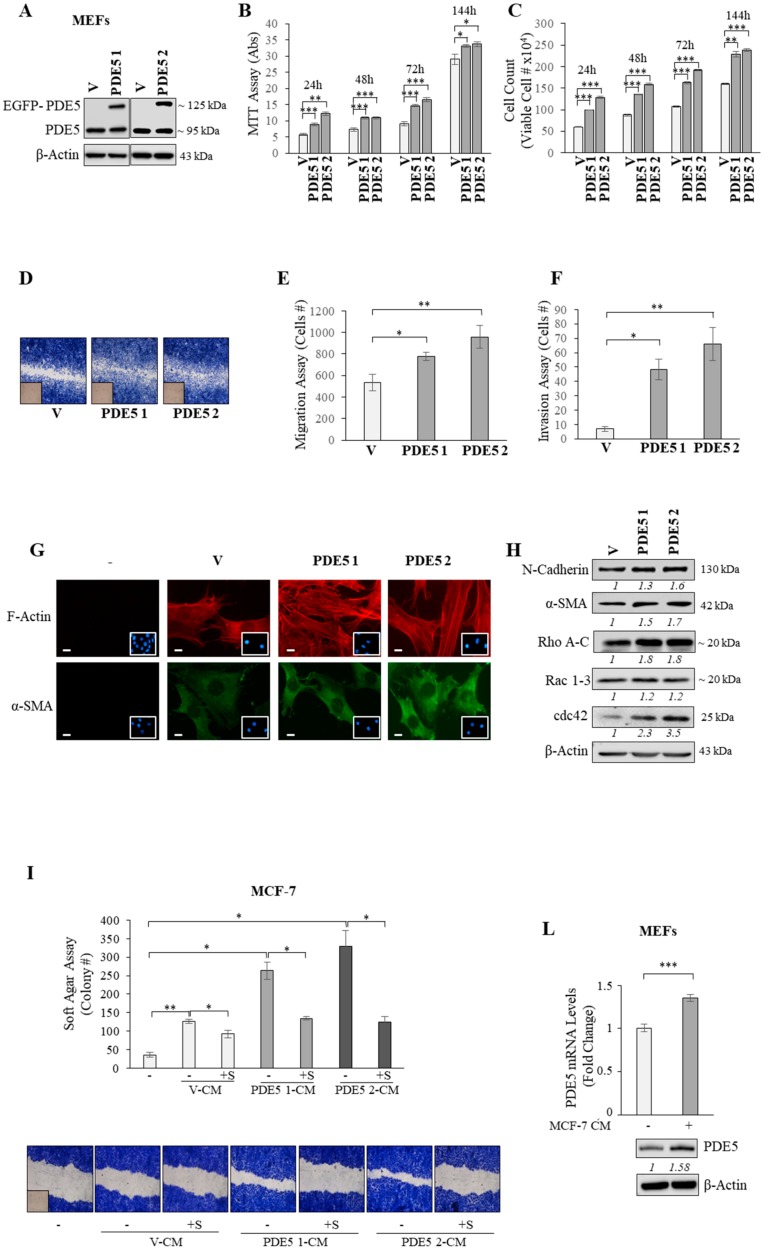
PDE5 overexpression and fibroblast activation. (**A**) Immunoblotting for PDE5 expression in mouse embryonic fibroblasts (MEFs) stably transfected with pEGFP (enhanced-green-fluorescent-protein) vector (V) and pEGFP-PDE5A expression plasmid (PDE5 1 and PDE5 2). β-Actin was used as a control for equal loading and transfer. Italicized numbers below blots represent the mean of the band optical density expressed as fold over V for PDE1 and PDE2. (**B**) MTT growth and (**C**) Trypan blue cell count assays in V, PDE5 1, and PDE5 2 stable clones under basal nonstimulated conditions at indicated times. (**D**) Wound healing assay in V, PDE5 1, and PDE5 2 stable clones with images captured at 0 (inset) and 12 h. Pictures are representative of three independent experiments. (**E**) Boyden chamber transmigration and (**F**) invasion assays in V, PDE5 1, and PDE5 2 stable clones under basal nonstimulated conditions. (**G**) Immunofluorescent staining of phalloidin staining of F-actin (stress fibers, red, upper panel) and α-SMA (lower panel) and in stable clones. 4′,6-Diamidino-2-phenylindole (DAPI) staining was used for nuclei detection (inset). Pictures are representative of three independent experiments. Scale bar = 5 µm. (**H**) Immunoblotting for N-cadherin, α-SMA, Rho A-C, Rac 1-3, and cdc42 expression levels in V, PDE5 1, and PDE5 2 stable clones. β-Actin was used as a control for equal loading and transfer. Italicized numbers below blots represent the mean of the band optical density expressed as fold over V for PDE1 and PDE2. (**I**) Soft agar growth (upper panel) and wound healing (lower panel) assays in MCF-7 breast cancer cells incubated with conditioned medium (CM) derived from V, PDE5 1, and PDE5 2 stable MEFs treated with vehicle (-) or sildenafil (+S, 10 μM). (**L**) Real-time RT-PCR assay and immunoblotting for PDE5 expression in MEFs treated for 24 h with control medium (-) or CM derived from MCF-7 breast cancer cells. β-Actin was used as a control for equal loading and transfer. Italicized numbers below blots represent the mean of the band optical density expressed as fold over MFEs treated with control medium (-) for MEFs treated with MCF-7 CM. The values represent the mean ±SEM of three different experiments, each performed in triplicate. * *p* < 0.05, ** *p*, < 0.005, *** *p* < 0.0005.

To better evaluate the significance of PDE5 overexpression in stroma, immortalized mouse embryonic fibroblasts (MEFs) were selected to have an experimental ‘in vitro’ cellular model displaying forced overexpression of this enzyme. PDE5 cDNA, cloned in frame with the enhanced-green-fluorescent-protein (EGFP) in mammalian pEGFP-C1 expression vector, was stably transfected in MEFs and obtained clones were examined by using immunoblotting analysis. The presence of endogenous (~95 kDa) and exogenous (EGFP-tagged, ~125 kDa) PDE5 bands in protein extracts from PDE5-overexpressing stable clones was shown (Figure 4A). First, we evaluated whether the overexpression of PDE5 may induce modifications in cellular phenotype, such as proliferative, motile, and invasive features of MEFs. MTT and cell count anchorage-dependent growth assays revealed an increase in cell proliferation in PDE5-overexpressing fibroblasts (PDE5 1 and 2) in comparison with control vector-expressing clones (V) (Figure 4B,C). Then, we assessed whether PDE5 overexpression may influence fibroblast motility in wound-healing assays and observed that PDE5-overexpressing cells moved faster than V clones (Figure 4D). Accordingly, we also found that PDE5 overexpression significantly increased both motility and invasion of MEFs in Boyden chamber transmigration and invasion assays, respectively (Figure 4E,F). We then evaluated whether PDE5 overexpression could affect actin organization, specifically actin stress-fiber formation that is crucial in the transmission of the cellular forces required for motility [29,30]. In vector-expressing fibroblasts, actin fibers were visible as bright structures diffusely distributed in the cytoplasm, while an increase of stress fiber formation was evident in PDE5-expressing MEFs (Figure 4G, upper panel). Accordingly, markers of fibroblast motility, including N-cadherin, α-SMA, Rho A-C, Rac 1-3, and cdc42, were up-regulated in PDE5 1 and 2 cells (Figure 4G, lower panel and 4H). After having evaluated the effects of PDE5 overexpression on fibroblast phenotype, we investigated whether or not PDE5 overexpression may impact tumor–stroma crosstalk in co-culture experiments. Soft agar assay showed that colony numbers of MCF-7 breast cancer cells were significantly increased when cells were incubated with CM derived from vehicle-treated PDE5-overexpressing MEFs compared to CM derived from vehicle-treated vector-expressing MEFs, while this induction was no longer evident when cells were co-cultured with CM derived from sildenafil-treated PDE5-overexpressing MEFs (Figure 4I, upper panel). As shown in Figure 4I (lower panel), PDE5 overexpression in MEFs also stimulated breast cancer cell motility and sildenafil treatment of CAFs reversed these effects, further highlighting the potential of PDE5 to influence stromal fibroblast activation.

To extend the results obtained, we generated pools of pEGFP-Vector (V P) and pEGFP-PDE5 (PDE5 P) stable transfectants in MEFs (Supplementary Figure S2A). As previously demonstrated, increased proliferative, migratory, and invasive capabilities of MEFs (Supplementary Figure S2B–F) along with elevated expression of CAF markers (Supplementary Figure S2G,H) were observed in the presence of PDE5 overexpression. Again, growth and migration of MCF-7 breast cancer cells were enhanced in the presence of CM derived from PDE5-overexpressing fibroblasts compared to vector pools and this increase was reversed in the presence of CM derived from sildenafil-treated PDE5 pools (Supplementary Figure S2I).

Some studies have reported that cancer epithelial cells can also shape tumor stroma [6,31,32]. Interestingly, when MEFs were co-cultured with MCF-7 breast cancer cells, they exhibited an up-regulation of PDE5 in terms of both mRNA and protein levels (Figure 4L), supporting the idea that the ‘host’ stroma talks to and coevolves with the malignant epithelium during progression [33].

### 2.3. CXCL16 Mediates the Tumor Promoting Effects of PDE5-Overexpressing Fibroblasts

**Figure 5:**
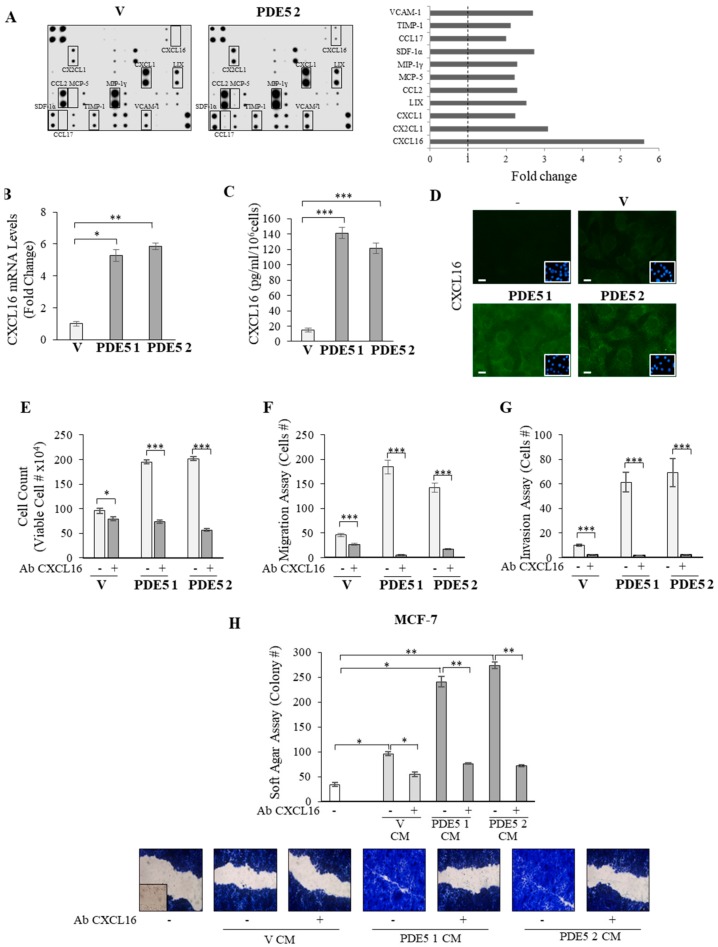
Role for C-X-C motif chemokine 16 (CXCL16) in mediating tumor-promoting features of PDE5-overexpressing fibroblasts. (**A**) Mouse cytokine arrays for the detection of secreted proteins in conditioned medium derived from vector (V) and PDE5-overexpressing (PDE5 2) MEFs. Left panels: membranes, 62 targets detected. Right panels: raw numerical densitometry data were extracted, the background subtracted, and the data normalized to the positive control signals. Results are shown as fold change of PDE5 2 versus V stable clones. (**B**) Real-time RT-PCR assay for CXCL16 mRNA expression in V, PDE5 1, and PDE5 2 stable clones. (**C**) ELISA for CXCL16 protein secretion in V, PDE5 1, and PDE5 2 stable clones. (**D**) Immunofluorescent staining of CXCL16 protein expression in V, PDE5 1, and PDE5 2 stable clones. DAPI staining was used for nuclei detection (inset). Scale bar = 5 µm. (**E**) Trypan blue cell count assays in V, PDE5 1, and PDE5 2 stable clones treated with vehicle (-) or CXCL16 blocking antibody (Ab CXCL16, 1.5 µg/mL) for 72 h. (**F**) Boyden chamber transmigration and (**G**) invasion assays in cells treated with vehicle (-) or Ab CXCL16. (**H**) Soft agar growth (upper panel) and wound healing (lower panel) assays in MCF-7 breast cancer cells exposed with conditioned medium (CM) derived from V, PDE5 1, and PDE5 2 stable MEFs treated with vehicle (-) or Ab CXCL16. Inset, time 0. Pictures are representative of three independent experiments. The values represent the mean ±SEM of three different experiments, each performed in triplicate. * *p* < 0.05; ** *p* < 0.005; *** *p* < 0.0005.

Activated fibroblasts secrete a number of signaling molecules that promote tumorigenesis [3,28,34]. Therefore, to gain more insights into the biological properties of PDE5-overexpressing fibroblasts, we analyzed factors secreted from vector (V) and PDE5-overexpressing (PDE5 2) clones by cytokine array (Figure 5A). The comparison of the secretome between the two cell models revealed differences in several secreted proteins, and among them, the chemokine CXCL16 was the most highly upregulated in response to PDE5 overexpression. To validate this expression profile, we first compared the mRNA levels of CXCL16 in vector and PDE5-transfected cells. As shown in Figure 5B, increased expression of this gene was detected in cloned PDE5-expressing fibroblasts compared with vector ones. Moreover, ELISA (Figure 5C) and immunofluorescent staining (Figure 5D) revealed higher protein expression levels of CXCL16 in PDE5-overexpressing fibroblasts than in vector MEFs. Consistent with these results, treatment with a neutralizing antibody to CXCL16 reduced proliferation (Figure 5E), motility (Figure 5F), and invasion (Figure 5G) of PDE5-overexpressing fibroblasts. More relevantly, treatment with the CXCL16 neutralizing antibody was able to significantly abolish the stimulatory effects exerted by CM derived from PDE5-overexpressing MEFs on both growth and motility of MCF-7 breast cancer cells (Figure 5H).

Collectively, these results strongly suggest that increased stromal PDE5 may influence fibroblast phenotype and their effects on breast cancer cell growth and motility through CXCL16.

### 2.4. Association of PDE5 and CXCL16 in the Stroma of Breast Cancer Patients

**Figure 6:**
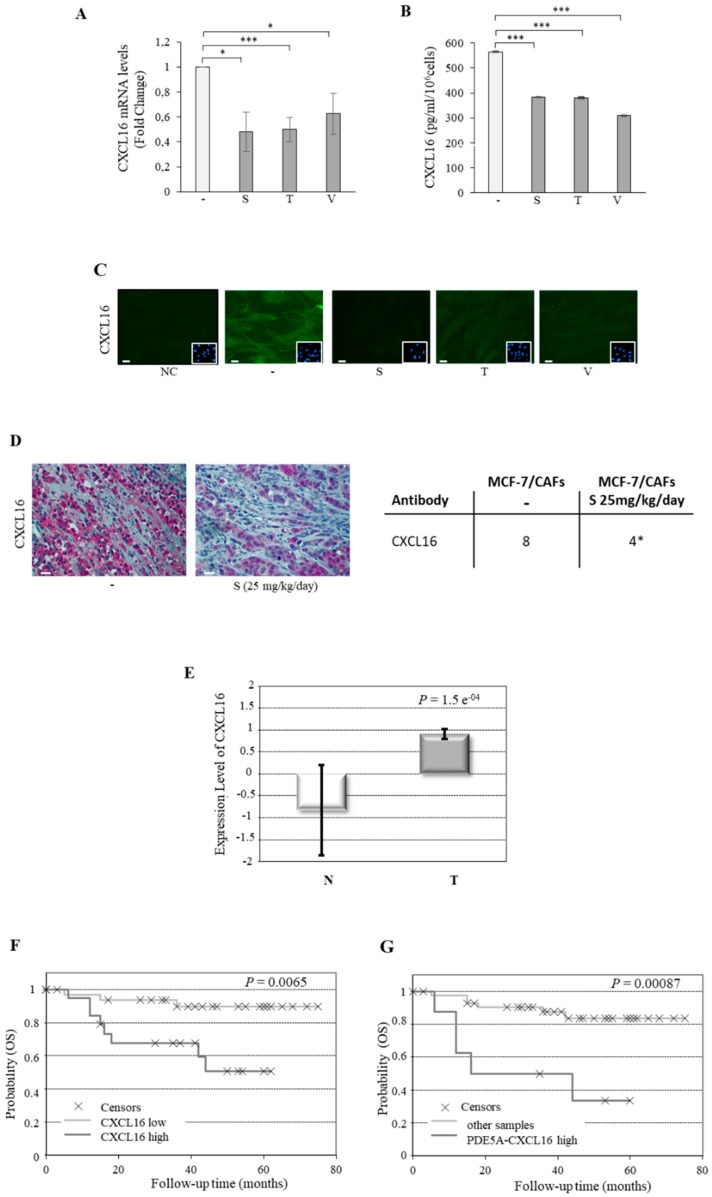
CXCL16 and PDE5 correlation in the stroma of breast cancer patients. (**A**) Real-time RT-PCR assay for CXCL16 mRNA expression in CAFs treated with vehicle (−) or the PDE5 inhibitors: sildenafil (S, 10 µM), tadalafil, (T, 10 µM), and vardenafil (V, 10 µM) for 24 h. (**B**) ELISA and (**C**) immunofluorescent staining for CXCL16 protein levels in CAFs treated as indicated for 24 h. DAPI staining was used for nuclei detection (inset). Scale bar = 5 µm. The values represent the mean ± SEM of three different experiments, each performed in triplicate. * *p* < 0.05; *** *p* < 0.0005. (**D**) Left panel: human CXCL16 immunohistochemical staining of MCF-7/CAF xenograft tumor sections. Scale bar = 25 µm. Right panel: immunohistochemistry scores. Cases were scored according to Allred immunohistochemistry (IHC) score [28] which includes both the proportion and intensity scores (range from 0 to 8). * *p* < 0.05. (**E**) Gene expression levels of CXCL16 in normal (N) and breast cancer (T) stroma samples. (**F**) Kaplan–Meier survival analysis relating stromal CXCL16 levels and overall survival (OS) in breast cancer patients. (**G**) Kaplan–Meier survival analysis relating high stromal PDE5-CXCL16 levels and OS in breast cancer patients.

In order to further examine the role of CXCL16 as a key player in PDE5-associated stromal activation, we evaluated changes in CXCL16 expression in human breast CAFs treated with PDE5 inhibitors. Real-time RT-PCR assay revealed that treatment with sildenafil, tadalafil, and vardenafil led to a significant reduction of CXCL16 mRNA levels (Figure 6A). In addition, both ELISA (Figure 6B) and immunofluorescent staining (Figure 6C) demonstrated that PDE5 inhibition reduced CXCL16 protein expression in CAFs. Accordingly, immunohistochemistry analysis in mouse tumor tissues showed that sildenafil decreased stromal CXCL16 levels also ‘in vivo’ (Figure 6D).

Finally, to support the clinical significance of these findings, we analyzed CXCL16 expression levels in the previously mentioned human breast tumor stroma microarray. CXCL16 expression was found to be higher in stroma samples of invasive ductal carcinomas compared to normal adjacent stroma (Figure 6E). The Kaplan–Meier overall survival curve indicated that increased expression levels of stroma CXCL16 are associated with a statistically significant shorter survival (Figure 6F). As previously shown for stromal PDE5, CXCL16 expression did not significantly correlate with breast cancer molecular subtypes, lymph node status, grade, ER, PR, and HER2 positivity. However, we found that CXCL16 showed a strong correlation with PDE5 expression in the same dataset (*p* = 0.00023). In addition, patients with high PDE5 and high CXCL16 levels had a statistically significantly poorer overall survival compared with all other patients (Figure 6G).

Therefore, high levels of PDE5 and CXCL16 can be detected in breast tumor stroma and their stromal expression may predict poor outcome among breast cancer patients.

## 3. Discussion

While the initial view was that PDE5 is highly expressed and can function in breast cancer cells, we show here that this enzyme is also upregulated in CAFs when compared to normal fibroblasts and this overexpression is a part of the dialogue between breast cancer cells and CAFs, allowing CAFs via CXCL16 secretion to promote breast cancer progression. These data shed new light on the mechanisms underlying the cross-talk of stromal-tumor cells and may hold prognostic information as well as a new strategy for stromal cell targeting.

Over a century ago, the seminal work of English surgeon Stephen Paget reported that cancer cells constitute the ‘seeds’ that colonize a receptive stromal microenvironment acting as a favorable ‘soil’. A crucial ‘soil’ compartment is represented by fibroblasts that acquire a cancerous phenotype in response to the ‘seed’ cancer cells to facilitate their proliferation, invasion, and metastasis. Thus, CAFs not only support, but are the key protagonists in breast carcinogenesis, owing to their abundance and their tumor-promoting roles [2,35]. However, the molecular genetic drivers governing the emergence of CAF cellular behavior and the regulation of stromal–epithelial interactions are still poorly understood. Some reports have shown that specific gene transcripts within CAFs may be involved in breast cancer cell growth and metastasis, such as the loss of p85α [32], the Phosphatase and tensin homolog (PTEN) [36], or TGF-receptor type 2 [37] as well as the overexpression of cyclin D1 [38]. Herein, we show that PDE5 overexpression may represent an important feature of fibroblast activation and heterotypic signaling promotion within the breast tumor microenvironment. Indeed, we found that the levels of PDE5 gene are increased in breast cancer stroma compared to normal stroma and this overexpression correlates with the worst survival outcomes, supporting the idea that stromal PDE5 may have an important effect on breast tumor growth. Indeed, stable overexpression of PDE5 activates normal fibroblasts towards a cancer-associated phenotype, as revealed by enhanced cell proliferative, migratory, and invasive capabilities along with the increased expression of activated fibroblast markers. PDE5 overexpression in fibroblasts also enhanced breast–tumor crosstalk in ‘co-culture’ experiments. The functional relevance of stromal PDE5 was highlighted by the inhibitory effects exerted by PDE5-targeting drugs on the activated features of breast CAFs and on breast tumor progression in both ‘in vitro’ and ‘in vivo’ experimental models. Of relevance, we found that normal fibroblasts co-cultured with breast cancer cells exhibited an increased PDE5 mRNA and protein expression, pointing out stromal PDE5 as a likely actor in the bidirectional crosstalk between the cancer bulk and its surrounding microenvironment.

Accumulating evidences suggest that a wide array of signaling molecules, importantly chemokines, stand at the crossroads of tumor–fibroblast interactions and subsequently impact most of the hallmark capabilities of breast cancer [34]. For instance, the C-X-C chemokine network represents a unique group of molecules and receptors recognized for their involvement in tumor biology, including their direct effects on cancer cells, such as transformation, survival, and proliferation, and indirect effects, such as angiogenesis and immune cell recruitment into tumor sites [3,28,34]. Several studies have proposed a multifaceted role for CXCL16/CXCR6 axis in the progression of different tumors, including those of the prostate, liver, ovaries, and breast [39,40,41,42,43,44], as was widely demonstrated for CXCL12 and its cognate receptor CXCR4 pair [3]. Indeed, CXCR6 and CXCL16 are upregulated in multiple cancer tissues, including primary and metastatic tissues, and cancer cell lines compared to respective normal ones and their levels increase as tumor malignancy progresses [39,40,41,42,43,44]. Moreover, CXCL16 via interaction with CXCR6 is capable of increasing cell migration, invasion, and metastasis in breast cancer [42,45]. Our findings indicated that PDE5 overexpression can activate stromal fibroblasts to produce and secrete CXCL16 that, in turn, may contribute to breast cancer progression by serving as a proliferative signal and as a regulator of motility. Accordingly, the addition of a CXCL16 neutralizing antibody abolished the pro-tumoral stimulatory effects of conditioned medium derived from PDE5-overexpressing fibroblasts on breast cancer cell growth and migration. However, fibroblast-secreted CXCL16 not only orchestrates paracrine pro-tumorigenic signaling to the adjacent breast cancer cells, but it can also modulate intrinsic characteristics of activated fibroblasts in an autocrine manner, since a significant inhibition in the proliferative, motile, and invasive capabilities of activated PDE5-overexpressing fibroblasts was evident in the presence of a CXCL16 neutralizing antibody. On the other hand, PDE5 inhibition significantly reduced CXCL16 expression and secretion in CAFs, indicating that CXCL16 and PDE5 levels can be correlated. Indeed, as shown for stromal PDE5 levels, increased stromal expression of CXCL16 was found in breast cancer stroma compared to the corresponding normal tissue stroma and was associated with poor overall survival in Kaplan–Meier analysis. More importantly, our results showed a significant association between the expression levels of PDE5 and CXCL16 in the stroma of breast cancer patients and a statistically significantly poorer overall survival in patients with high PDE5 and high CXCL16 levels compared with all other patients.

## 4. Materials and Methods

### 4.1. Reagents, Antibodies, and Plasmids

Sildenafil citrate (s1431), tadalafil (s1512), and vardenafil hydrochloride trihydrate (s2515) were from Selleckchem (Munich, DE, Germany). Neutralizing antibody to CXCL16 (MBA503) was from R&D Systems (Minneapolis, MN, USA). Antibodies were directed to PDE5A (sc-32884, SantaCruz Biotechnology (Dallas, TX, USA), 1:500), β-actin (sc-69879 SantaCruz Biotechnology, 1:5000), α-SMA (a5228, Sigma Aldrich (Milano, Italy, IT), 1:1000 for immunoblot analysis, 1:100 for fluorescence microscopy, and 1:50 for immunohistochemical analysis), N-cadherin (sc-271386, SantaCruz Biotechnology, 1:500), RhoA/RhoB/RhoC (MA1-011, Thermo Fisher Scientific (Waltham, MA USA), 1:500), Rac1/Rac2/Rac3 (PA5-17519, Thermo Fisher Scientific, 1:1000), Cdc42 (sc-8401, SantaCruz Biotechnology, 1:500), Ki-67 (MIB-1, Dako Denmark (Santa Clara, CA, USA), 1:100), CK18 (sc-51583, SantaCruz Biotechnology, 1:100), mouse CXCL16 (ab119350, Abcam (Cambridge, UK, Britain), 1:100), and human CXCL16 (ab101404, Abcam, 1:150). pEGFP-vector and pEGFP-PDE5A were kindly provided by F. Barbagallo (Sapienza University, Rome, Italy).

### 4.2. Isolation and Culture of Cancer-Associated Fibroblasts and Normal Fibroblasts

Establishment of human breast fibroblast cells from fresh tumors (CAFs) and normal tissues (NFs) were obtained from women who signed informed consent, following previously described procedures [46]. Cells were cultured in RPMI-1640 medium supplemented with 15% fetal bovine serum (FBS) and antibiotics, tested by mycoplasma presence (MycoAlert Mycoplasma Detection Assay, Lonza, Basilea, CH, Svizzera), and characterized as shown in Supplementary Figure S1. All experiments were performed before the 10th passage. These study was approved by the Ethic Institutional Committees at Annunziata Hospital, Cosenza, Italy (#149 issued by Comitato Etico Regione Calabria, Sezione Area Nord c/o Azienza Ospedaliera di Cosenza, 28/10/2015). Experimental procedures for fibroblast isolation were performed in accordance with approved guidelines.

### 4.3. Cell Cultures

Mouse embryonic fibroblasts (MEFs) and MCF-7 breast cancer cell lines were from American Type Culture Collection. All cell lines, stored and authenticated following suppliers, were used within six months after frozen aliquot resuscitations and were regularly tested for mycoplasma-negativity (MycoAlert Mycoplasma Detection Assay). To generate PDE5A-overexpressing MEFs, cells were transfected with pEGFP-vector or pEGFP-PDE5A vector (5 µg/10 cm dishes) using Fugene 6 reagent, following the manufacturer’s instructions (Promega, Madison, WI, USA). Stable clones were selected with G418 antibiotic (1 mg/mL, Thermo Fisher Scientific). PDE5-expressing MEF pools stably transfected with pEGFP-vector or pEGFP-PDE5A vectors were also used. Positive clones were identified using immunoblot analysis.

### 4.4. Conditioned Medium Systems

CAFs or MEF stable clones were treated as indicated in the respective experiments for 8–12 h. Then, cells were washed twice and cultured with 3% charcoal-stripped serum medium for 36 h. In another set of experiments, MCF-7 breast cancer cells were cultured with 3% charcoal-stripped serum media for 24 h. Conditioned medium (CM) was collected, centrifuged, and used in co-culture experiments.

### 4.5. Real-Time RT-PCR Assays

FAP, α-SMA, PDE5, and CXCL16 gene expression was assessed by real-time reverse transcription (RT)-PCR, using SYBR Green Universal PCR Master Mix (Bio-Rad, Hercules, CA, USA). Each sample was normalized on 36B4 or GAPDH (Glyceraldehyde-3-Phosphate Dehydrogenase) content, according to its mouse or human origin. Relative gene expression levels were calculated as previously described [47]. Primers used are listed in Supplementary Table S1.

### 4.6. Immunoblot Analysis

Cell extracts were resolved by SDS-PAGE, as described in [48]. Immunoblots show a single representative of three separate experiments. Images were acquired using Odissey FC (Licor, Lincoln, NB, USA).

### 4.7. Cell Proliferation Assays

Trypan Blue Cell Count Assays. Cell numbers were evaluated by trypsin suspension of samples using a hemocytometer as described [49].

MTT Assays. Cell proliferation was assessed by 3-(4,5-Dimethylthiazol-2-yl)-2,5-diphenyltetrazolium bromide reagent (MTT, Sigma Aldrich) as described [50].

Soft Agar Growth Assays. Anchorage-independent growth assays were performed as described [50].

Data represent three independent experiments performed in triplicate.

### 4.8. Wound Healing Assays

Cell monolayers were scraped and exposed to the following experimental conditions: (i) CAFs were treated with sildenafil, tadalafil, or vardenafil (10 µM); (ii) vector and PDE5 stable pools or stable clones were left under basal nonstimulated conditions; (iii) MCF-7 cells were treated with control medium or conditioned medium derived from CAFs exposed to vehicle, sildenafil, tadalafil, or vardenafil (10 µM) or conditioned medium derived from vector and PDE5-overexpressing stable clones exposed to vehicle or sildenafil (10 µM) or CXCL16 blocking antibody (1.5 µg/mL) or conditioned medium derived from vector and PDE5-overexpressing stable pools exposed to vehicle or sildenafil (10 µM). Wound closure was monitored over 8–12 hours, cells were fixed and stained with Coomassie Brillant Blue. Pictures represent one of three independent experiments.

### 4.9. Boyden Chamber Transmigration Assays

CAFs treated with sildenafil, tadalafil, or vardenafil (10 µM) were placed in the upper compartments of Boyden chambers (8 µm-membranes, Corning). In another set of experiments, vector and PDE5-overexpressing stable pools or stable clones left under basal nonstimulated conditions and vector and PDE5 stable clones exposed to vehicle or CXCL16 blocking antibody (1.5 µg/mL) were placed in the upper compartments of Boyden chambers. Bottom wells contained regular growth media. After 8–12 h, migrated cells were fixed and stained with DAPI. Migration was quantified by viewing six separate fields per membrane at 10× magnification (OLYMPUS-BX51 microscope) and expressed as mean numbers of migrated cells. Data represent three independent experiments, each assayed in triplicate.

### 4.10. Boyden Chamber Invasion Assays

Matrigel-based invasion assay was performed in chambers (8 µm-membranes, Corning) coated with Matrigel (BD Bioscences, 2 mg/mL). Cells under the same experimental conditions indicated in Section 4.9 were seeded into top transwell-chambers and regular growth medium was used in the lower chambers. After 8–12 h, invaded cells were quantified as reported for transmigration assays. Data represent three independent experiments, each assayed in triplicate.

### 4.11. Fluorescence Microscopy

Cells were fixed with 4% paraformaldehyde and permeabilized with PBS +0.2% Triton X-100 and immunofluorescent staining was performed as previously described [51]. 4′,6-Diamidino-2-phenylindole (DAPI, Sigma) staining was used for nuclei detection. Fluorescence was photographed at 100× magnification (OLYMPUS-BX51 microscope, Segrate, IT, Italy).

### 4.12. Phalloidin Staining

Polymerized actin stress fibers were stained with Alexa Fluor 568-conjugated phalloidin (1:500), following manufacturer’s instructions (Thermo Fisher Scientific). Cell nuclei were counterstained with DAPI. Olympus BX51 microscope (100× magnification) was used for imaging.

### 4.13. Mouse Cytokine Array

MEF stable clones were cultured with 3% charcoal-stripped serum media for 36 h. CM was collected, centrifuged, and used in mouse cytokine arrays (AAM-CYT-3, Raybiotech), as described in the manufacturer’s protocol.

### 4.14. CXCL16 Measurement by Enzyme-Linked Immunosorbent Assay (ELISA)

Mouse CXCL16 was measured in MEF CM by using RayBio Mouse CXCL16 ELISA Kit based on the manufacturer’s protocol (RayBiotech, Peachtree Corners, GA, USA). Human CXCL16 was measured in CAF CM by using RayBio Human CXCL16 ELISA Kit based on the manufacturer’s protocol (RayBiotech). Results are presented as pg/mL/number of cells.

### 4.15. ‘In Vivo’ Experiments

The ‘in vivo’ experiments were performed in 45-day-old female athymic nude mice (Harlan Laboratories, Udine, Italy) maintained in a sterile environment. The animals were fully anesthetized by intraperitoneal (i.p.) injection of avertin 250 mg/kg to allow the subcutaneous implantation of estradiol (E_2_) pellets (0.72 mg per pellet, 90-day release; Innovative Research of America) into the dorso-lateral region. The day after, exponentially growing MCF-7 cells (5.0 × 10^6^ cells/mouse) in combination with CAFs (ratio 3:1 tumor cells/CAFs) were inoculated subcutaneously in 0.1 mL of Matrigel into the intrascapular region. The subcutaneous location was chosen to exclude the effect of other mammary gland cellular components on the interaction of human breast cancer fibroblasts and epithelial cells. Sildenafil treatment (25 mg/kg/day) was started when mean tumor size reached 150 mm^3^ (day 0) and was delivered daily to the animals by i.p. injection. Tumor development was followed twice a week by caliper measurements along two orthogonal axes: length (L) and width (W). The volume (V) of tumors was estimated by the following formula: V = L × (W^2^)/2. At day 20 the animals were sacrificed following the standard protocols and tumors were dissected from the neighboring connective tissue. Specimens of tumors were frozen in nitrogen and stored at –80 °C. The remaining tumor tissues of each sample, livers, lungs, spleens, and kidneys were fixed in 4% paraformaldehyde and embedded in paraffin for the histological analyses. All animals were maintained and handled in accordance with the recommendation of the Guidelines for the Care and Use of Laboratory Animals and experiments were approved by the Animal Care Committee of University of Calabria, Italy (1290/2015-PR, 18/12/2015).

### 4.16. Histopathological Analysis

Tumors, livers, lungs, spleens, and kidneys were fixed in 4% formalin, sectioned into 5 μm of thickness and stained with hematoxylin and eosin Y (H&E), as suggested by the manufacturer (Bio-Optica). H&E was photographed at 20× magnification (Olympus BX51 microscope, Segrate, IT, Italy).

### 4.17. Immunohistochemical Analysis 

Paraffin-embedded sections, 5 μm thick, were mounted on slides precoated with poly-lysine, and then they were deparaffinized and dehydrated (seven to eight serial sections). Immunohistochemical experiments were performed after heat-mediated antigen retrieval, using human Cytokeratin 18, α-SMA, Ki-67, or CXCL16 primary antibodies at 4 °C overnight. Then, a biotinylated specific IgG was applied for 1 h at room temperature, followed by the avidin biotin alkaline phosphatase complex (VECTASTAIN^®^ ABC-AP KIT, Vector Laboratories, Burlingame, CA, USA). Immunoreactivity was visualized by using VECTOR^®^ Red Alkaline Phosphatase Substrate Kit (Vector Laboratories). Counterstaining was carried out with hematoxylin (Sigma Aldrich). The primary antibody was replaced by normal serum in negative control sections. The immunostained slides of tumor samples were evaluated by light microscopy using the Allred score [52], which combines a proportion score and an intensity score. A proportion score was assigned representing the estimated proportion of positively stained tumor cells (0 = none; 1 = 1/100; 2 = 1/100 to <1/10; 3 = 1/10 to <1/3; 4 = 1/3 to 2/3; 5 = >2/3). An intensity score was assigned by the average estimated intensity of staining in positive cells (0 = none; 1 = weak; 2 = moderate; 3 = strong). Proportion score and intensity score were added to obtain a total score that ranged from 0 to 8. A minimum of 100 cells were evaluated in each slide. Six to seven serial sections were scored in a blinded manner for each sample. The one-way ANOVA was used to evaluate the differences in the scores between tumor and control samples.

### 4.18. Analysis of Human Stroma Gene Expression Data

The data regarding human breast cancer stroma-based gene expression (GSE9014) were downloaded from the the Gene Expression Omnibus (GEO) repository at the National Center for Biotechnology Information (NCBI) [53]. Briefly, from a cohort of 73 women with invasive breast carcinoma diagnosis (median follow-up time: 3.58 years), 53 samples of tumor stroma and 31 individual-matched normal adjacent stroma samples were collected. ER positive (luminal) samples constituted 72.3% of the patients, HER2 enriched (HER2 positive ER negative) constituted 10.3% of the specimens, and triple negative (ER/PR/HER2 negative) constituted 11.8% of the patients. The remaining patients (5.6%) had discordant status for ER and PR determination. No woman in the study was treated with neoadjuvant therapy. Poor outcome was defined as alive with disease or dead of disease at the time of the latest follow-up. This dataset was generated using the Agilent-012391 Whole Human Genome Oligo Microarray G4112A platform. The probe 37097 was used to detect PDE5 expression. The dye-swapped repetitions were inverted and averaged to get the final expression value for each sample.

### 4.19. Statistical Analysis

Data were analyzed for statistical significance using two-tailed Student’s t-test, and GraphPad-Prism 4 software program. SEM is shown. Kaplan–Meier survival plot and log rank *p*-value were calculated and plotted in R as described [54]. Cox proportional hazard regression was computed to compare the association between gene expression, clinical variables including ER/PR/HER2/lymph node/grade status, and survival in multivariate analysis using WinSTAT 2014 for Microsoft Excel (WinStat 2014, Robert K. Fitch Software, Bad Krozingen, DE, Germany). Statistical significance was set at *p* < 0.05.

## 5. Conclusions

Breast tumor microenvironment represents a functional ecosystem of neoplastic epithelial cells and stroma with critical roles in most of cancer progression processes. Therefore, it is not surprising that an increasing proportion of research is currently focusing on the development of double-acting anti-cancer strategies targeted toward both breast cancer cells and tumor microenvironment. Another more pragmatic reason underlying the exciting interest on anti-stromal therapies is that stromal cells are more genetically stable than cancer cells [55]. In light of the present and our previous study [14], we can speculate that PDE5 activity may regulate several aspects of breast carcinogenesis both directly on neoplastic epithelial cells and indirectly via modulation of CAF phenotype. Therefore, since PDE5 inhibitors are safely and successfully used in patients suffering from different pathologies [56,57], our results may endorse their use as repurposed concurrent treatments to conventional anti-cancer agents to provide more effective options for breast cancer women. To date, a randomized Phase I trial was completed for evaluating the potential of sildenafil to enhance doxorubicin anti-tumor effects and cardioprotection in breast cancer patients (NCT01375699), but no details have been published yet. In addition, stromal PDE5 may represent not only a feasible therapeutic target but may also hold a potential prognostic value identifying patients with a poorer clinical outcome. Taken together, the present findings may open a promising research area which needs to be further explored for deepening our understanding of tumor evolution and offering new avenue for future cancer management.

## Supplementary Materials

The following are available online at https://www.mdpi.com/2072-6694/11/11/1740/s1. Figure S1: Characterization of human CAFs; Figure S2: Fibroblasts activation after PDE5 overexpression in stable pools; Figure S3: Uncropped Western blots from primary figures are shown; Table S1: Primers used in this study.
