# Supplementary material for: Phosphodiesterase 5 (PDE5) Is Highly Expressed in Cancer-Associated Fibroblasts and Enhances Breast Tumor Progression
Source: Cancers (Basel). 2019 Nov 6;11(11):1740. doi: 10.3390/cancers11111740 (PMC6895904; doi:10.3390/cancers11111740)
Supplement: Supplementary file 1 [file cancers-11-01740-s001.pdf]

cancers

MDPI

Article

Phosphodiesterase 5 (PDE5) is Highly Expressed in Cancer-Associated Fibroblasts and Enhances Breast Tumor Progression

Stefania Catalano [1], Salvatore Panza [1], Giuseppina Augimeri [1], Cinzia Giordano [1,2], Rocco Malivindi [1], Luca Gelsomino [1], Stefania Marsico [1], Francesca Giordano [1], Balázs Győrffy [3], Daniela Bonofiglio [1], Sebastiano Andò [1,2,†] and Ines Barone [1,†,*]

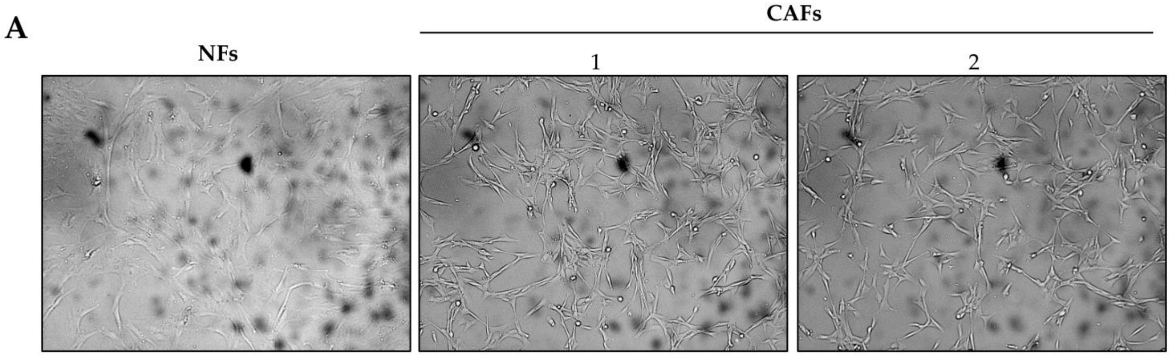

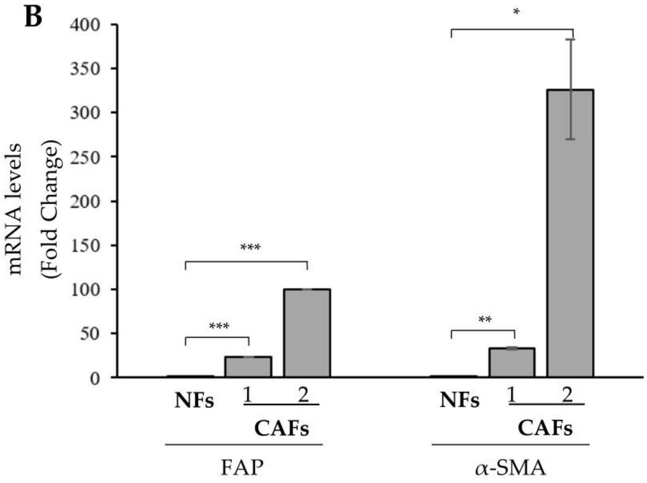

Supplementary Figure S1: Characterization of human CAFs. (**A**) Morphology of Normal Fibroblasts (NFs) and Cancer-associated Fibroblasts (CAFs1 and 2) in monolayer growth using phase contrast microscopy. (**B**) Realtime RT-PCR assay for FAP and α-SMA mRNA expression in NFs, CAFs1 and CAFs2. * $p < 0.05$, ** $p < 0.00ss5$, *** $p < 0.0005$.

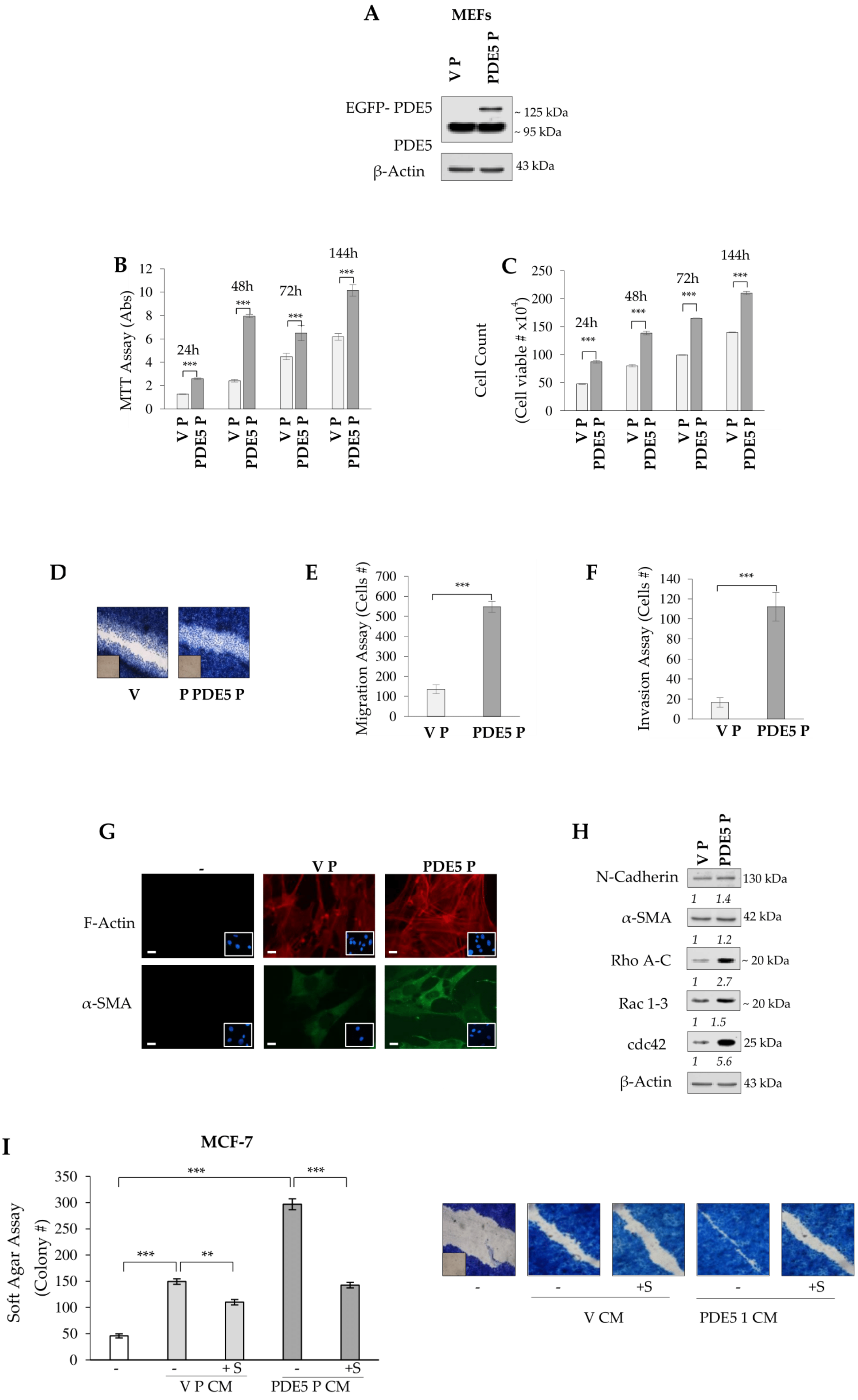

Supplementary Figure S2. (**A**) Immunoblotting for PDE5 expression in Mouse Embryonic Fibroblasts (MEFs) Vector (V P) and PDE5 (PDE5 P) stable pools. β-Actin was used as a control for equal loading and transfer. Italicized numbers below blots represent the mean of the band optical density expressed

as fold over V P for PDE P. MTT growth (**B**) and Trypan blue cell count (**C**) assays in V P and PDE5 P under basal nonstimulated conditions at indicated times. (**D**), Wound healing assay in V P and PDE5 P with images captured at 0 (*inset*) and 12 h. Pictures are representative of three independent experiments. Boyden-Chamber Transmigration (**E**) and Invasion (**F**) assays in V P and PDE5 P under basal nonstimulated conditions. (**G**), Immunofluorescent staining of Phalloidin staining of F-Actin (stress fibers, red, *upper panel*) α-SMA (*lower panel*) in stable pools. DAPI staining was used for nuclei detection (*inset*). Pictures are representative of three independent experiments. Scale bar = 5 μm. (**H**), Immunoblotting for N-Cadherin, α-SMA, Rho A-C, Rac 1-3, cdc42 expression levels in V P and PDE5 P. β-Actin was used as a control for equal loading and transfer. Italicized numbers below blots represent the mean of the band optical density expressed as fold over V P for PDE P. (**I**), Soft agar growth (*left panel*) and wound healing (*right panel*) assays in MCF-7 breast cancer cells incubated with conditioned medium (CM) derived from V P and PDE5 P treated with vehicle (-) or Sildenafil (+S, 10 μM). The values represent the mean ± S.E.M. of three different experiments, each performed in triplicate. * $p < 0.05$, ** $p < 0.005$, *** $p < 0.0005$.

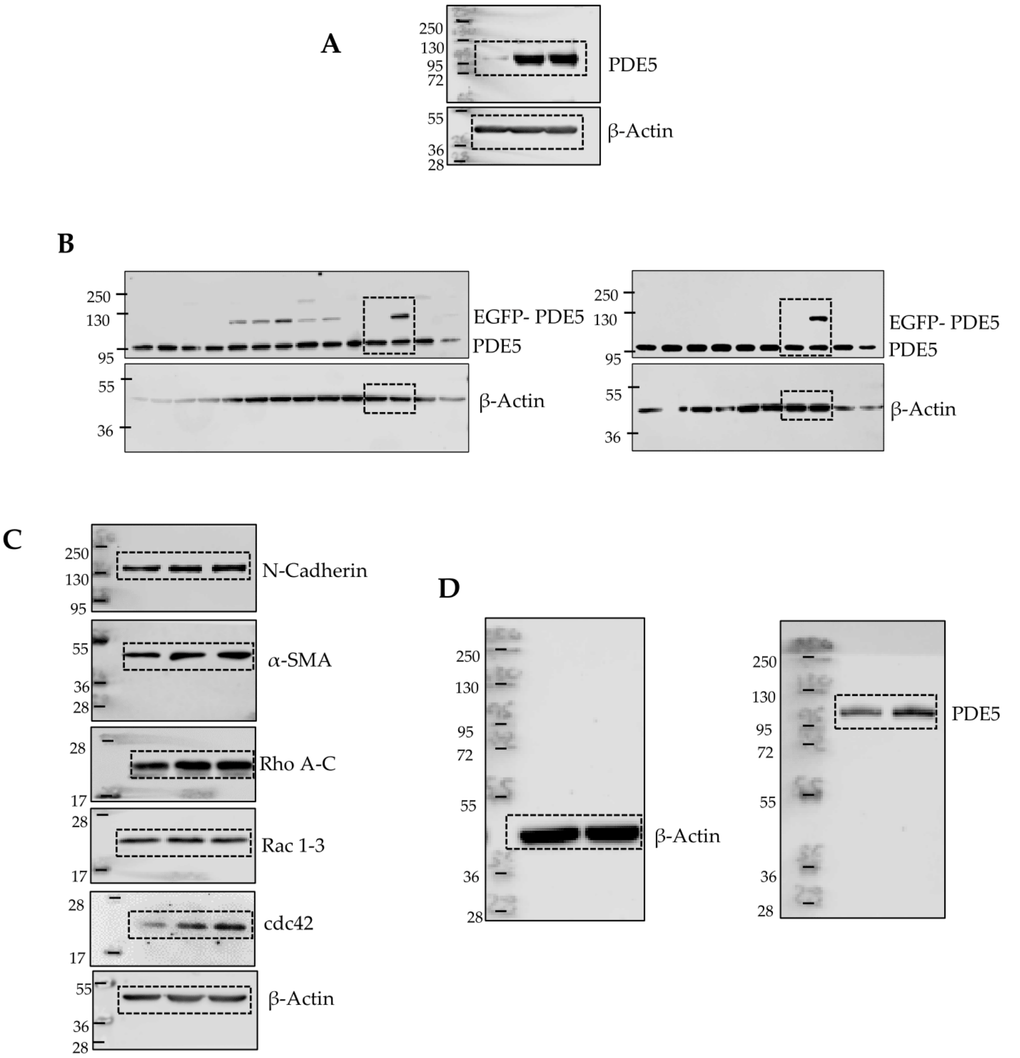

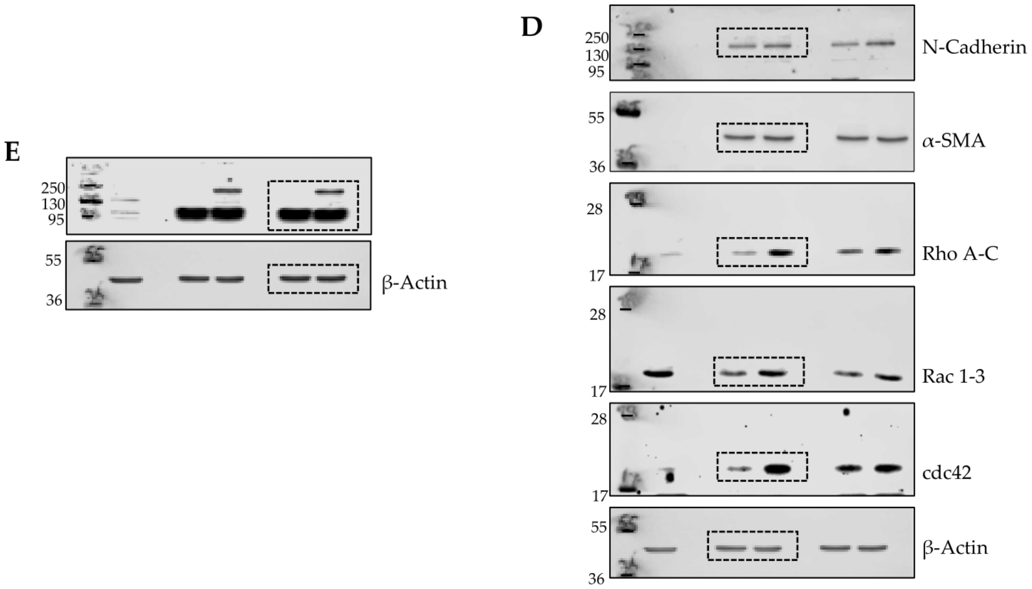

Supplementary Figure S3. Uncropped western blots from primary figures are shown. (**A**) Figure 2B; (**B**), Figure 4A; (**C**), Figure 4H; (**D**), Figure 4L; (**E**), Supplementary Figure 2A; (**F**), Supplementary Figure 2H.

Supplementary Table S1. Primers used in this study.

Gene Name	Gene Symbol	Species		Primer sequences
Alpha-Smooth Muscle Actin	*α-SMA*	*Human*	Forward	5′-GCAGCCCAGCCAAGCACTGT-3′
			Reverse	5′-TGGGAGCATCGTCCCCAGCA-3′
Fibroblast Activation Protein	*FAP*	*Human*	Forward	5′-AGAAAGCAGAACTGGATGG-3′
			Reverse	5′-ACACACTTCTTGCTTGGAGGAT-3′
Phosphodiesterase 5A	*PDE5A*	*Human*	Forward	5′-ACTTGCATTGCTGATTGCTG-3′
			Reverse	5′-TTGAATAGGCCAGGGTTTTG-3′
Phosphodiesterase 5A	*PDE5A*	*Mouse*	Forward	5′-GCGAGTGCAAGTTGAGTCTT-3′
			Reverse	5′-GCAGGAAGACGTTCACAGAC-3′
C-X-C motif chemokine Ligand 16	*CXCL16*	*Human*	Forward	5′-TCCAGATCTGCCGGTTCATT-3′
			Reverse	5′-GGGCAACATAGAGTCCGTCT-3′
C-X-C motif chemokine Ligand 16	*CXCL16*	*Mouse*	Forward	5′-ACTCAGCACTCCACTCTTCC-3′
			Reverse	5′-TCTCATTTGCCTCAGCCTCA-3′
Glyceraldehyde-3-Phosphate Dehydrogenase	*GAPDH*	*Human*	Forward	5′-TGGTATCGTGGAAGGACTCATGAC-3′
			Reverse	5′-ATGCCAGTGAGCTTCCCGTTCAGC-3′
36B4	*36B4*	*Mouse*	Forward	5′-AGATTCGGGATATGCTGTTGG-3′
			Reverse	5′-AAAGCCTGGAAGAAGGAGGTG-3′

SS
